# Short versus prolonged dual antiplatelet therapy (DAPT) duration after coronary stent implantation: A comparison between the DAPT study and 9 other trials evaluating DAPT duration

**DOI:** 10.1371/journal.pone.0174502

**Published:** 2017-09-20

**Authors:** Toshiaki Toyota, Hiroki Shiomi, Takeshi Morimoto, Masahiro Natsuaki, Takeshi Kimura

**Affiliations:** 1 Department of Cardiovascular Medicine, Kyoto University Graduate School of Medicine, Kyoto, Kyoto, Japan; 2 Department of Clinical Epidemiology, Hyogo College of Medicine, Nishinomiya, Hyogo, Japan; 3 Division of Cardiology, Saiseikai Fukuoka General Hospital, Fukuoka, Fukuoka, Japan; UMCU, NETHERLANDS

## Abstract

**Aims:**

The Dual Antiplatelet Therapy (DAPT) study demonstrated that DAPT beyond 1-year after drug-eluting stent (DES) implantation, as compared with aspirin therapy alone, significantly reduced the risk of major cardiovascular and cerebrovascular events, which was mainly driven by the large risk reduction for myocardial infarction (MI). We sought to compare the largest DAPT study with other trials evaluating DAPT durations after DES implantation.

**Methods and results:**

By a systematic literature search, we identified 9 trials comparing prolonged- versus short-DAPT in addition to the DAPT study. The result from the DAPT study (N = 9961) with public–private collaboration was different from the pooled result of the 9 other investigator-driven trials (N = 22174) in terms of the effect of prolonged-DAPT on MI (odds ratio [OR] 0.48 [95%CI 0.38–0.62] versus pooled OR 0.88 [95%CI 0.67–1.15]: P = 0.001 for difference), while the trends for excess risk of prolonged-DAPT relative to short-DAPT for all-cause death (OR 1.31 [95%CI 0.97–1.78] versus pooled OR 1.16 [95%CI 0.92–1.45]: P = 0.53 for difference), and bleeding (OR 1.62 [95%CI 1.21–2.17] versus pooled OR 2.08 [95%CI 1.51–2.84]: P = 0.25 for difference) were consistently seen in both the DAPT and other trials. The annual rate of MI during aspirin mono-therapy in the DAPT study was much higher than that those in the other trials (2.7% versus 0.6–1.6%).

**Conclusions:**

Given the difference between the DAPT study and other trials, future studies should focus on certain subgroups of patients that are more or less likely to benefit from longer duration DAPT.

## Introduction

The 2011 ACC/AHA/SCAI Guideline for Percutaneous Coronary Intervention recommended dual antiplatelet therapy (DAPT) consisting of aspirin and P2Y_12_-receptor inhibitor for at least 12 months after drug-eluting stent (DES) implantation.[1] However, a recent meta-analysis of trials comparing short- (< = 6-month) versus prolonged-DAPT (12-month or longer) duration suggested that extension of DAPT beyond 6-month increased the risk of bleeding without reducing ischemic events.[2] Based on these recent trial results, 2014 ESC/EACTS Guidelines on myocardial revascularization recommended DAPT for 6 months after new generation DES in stable coronary artery disease.[3] Recently, however, the Dual Antiplatelet Therapy (DAPT) study, a large international, multicenter, randomized, placebo-controlled trial, demonstrated that DAPT beyond 1-year after placement of a DES, as compared with aspirin therapy alone, significantly reduced the risks of stent thrombosis (ST) and major cardiovascular and cerebrovascular events.[4] The observation from the DAPT study was different from the findings in the previously reported trials comparing short- versus prolonged-DAPT duration, and has produced controversy regarding the optimal duration of DAPT after coronary stent implantation.[2]

Therefore, we conducted a systematic review of the trials comparing short- versus prolonged-DAPT duration after coronary stent implantation in an attempt to address the potential reasons for the discrepancy between the DAPT study and other trials.

## Materials and methods

We first conducted a systematic review and meta-analyses to obtain pooled odds ratio (OR) of prolonged-DAPT versus short-DAPT for clinically relevant outcomes among studies other than DAPT study. We then compared the pooled ORs between those of the DAPT study and the other trials.

### Search strategy and study identification

We searched all reported trials comparing DAPT duration in patients after coronary stent implantation, using the term "dual antiplatelet therapy", "DAPT", "aspirin", "clopidogrel", "prasugrel", "ticagrelor", "coronary intervention", "PCI", "stent", and "angioplasty". We searched the Pubmed, the United States National Institutes of Health clinical trials registry, and the Cochrane Central Register of Controlled trials. Additional search was performed using conference proceedings from the American College of Cardiology, the American Heart Association, the European Society of Cardiology, the Trans-catheter Cardiovascular Therapeutics, and Euro PCR meetings. Current analysis was limited to those studies including the participants with DES implantation. From the gathered studies, randomized controlled trials (RCT) comparing the effect of DAPT duration were extracted, and if there were several articles from the same RCT, we selected the article providing the longest follow-up data for the trial. The last search was performed in December 2014. Each trial was evaluated by referring to the Cochrane Collaboration’s tool for the adequacy of sequence generation, allocation concealment, blind assessment of participants and personnel, data reporting, and the other sources of bias.[5] Because this study used only published paper without individual patient information, the procedure of informed consent and institutional review board approval was not applicable.

### Statistical analysis

We collected baseline characteristics from each reported RCT. The risk estimates for each event of interest from individual reported RCT were gathered. The endpoints included, all-cause death, cardiac death, non-cardiac death, myocardial infarction (MI), ST, stroke, and bleeding. We used the outcome data after the landmark point, when patients started to be treated on each randomized regimen (aspirin mono-therapy, or DAPT). For the trials in which the risk estimates beyond the landmark point were not available, we substituted the reported risk estimates during the entire follow-up period. We used reported absolute number of patients with at least 1 event to calculate the risk estimates for each trial, and in case the absolute numbers were not reported, we estimated the number of patients from the reported cumulative incidence and the number of patients assigned to each group. We used the reported Kaplan-Meier curve to count or to read the cumulative incidence of outcome, if neither the absolute number nor the cumulative incidence for the outcome of interest were reported. We excluded the trials in which the event of interest was not observed in either study group, from the analysis for that event.

To assess the heterogeneity among the trials, we used the Cochrane test and calculated the I-square statistic for quantification, with values <25% indicating low, 25% to 50% indicating moderate, and >50% indicating high heterogeneity.[6] We used the Mantel-Haenszel method for the fixed-effect model for the calculations of pooled OR, unless heterogeneity exceeded moderate in each outcome. Publication bias was assessed by Egger’s test and Begg’s funnel plot.

We then compared ORs from the DAPT study and pooled ORs from meta-analyses of the trials other than the DAPT study by using the logarithmic transformed ORs and their standard errors, and obtaining the p-values for the null hypothesis that Ln (ORs of the DAPT study)–Ln (pooled ORs) was equal to zero. We also evaluated the annual rates of endpoint events on each randomized regimen if the definitions for the endpoint events were comparable.

The results were regarded as statistically significant at 2-sided P<0.05. Statistical analysis was performed using Stata software, version 13.1 (Stata Corp, College Station, TX).

## Results

### Search results

From the database search, we identified the DAPT study and 1018 records for the comparison of DAPT duration, and after the detailed evaluation including additional conference search, 10 RCTs were selected for the current analysis (S1 Table). All trials were evaluated along with the meta-analysis method and judged to have enough quality for the current analysis (S2 Table). The selection of studies was conducted according to the PRISMA (Preferred Reporting Items for Systematic reviews and Meta-Analysis) statement (S1 Fig).

### Characteristics of included trials

Among the 10 trials included in the current analysis, the DAPT study was designed in response to a request from the Food and Drug Administration (FDA) to manufacturers of coronary stents and was conducted under an investigational-device exemption through a public–private collaboration involving the FDA, eight stent and pharmaceutical manufacturers who funded the study, and the Harvard Clinical Research Institute (HCRI). Collection of data for the 5 separate studies that contributed to the DAPT study, was conducted by HCRI and 4 stent manufacturers (Abbott Vascular, Boston Scientific Corporation, Cordis Corporation, and Medtronic, Inc.). All the other 9 trials included in the current analysis were the investigator-driven trials. The DAPT study and ISAR-SAFE trial adopted double blind placebo-controlled design, while the other 8 trials took open-label design.

Age, gender, and prevalence of diabetes were comparable across the trials. Patients with acute coronary syndrome presentation at the index stent implantation ranged from 23% to 74%. Types of stents used were variable across the trials. In the DAPT study, 35% of patients received prasugrel as the P2Y_12_-receptor inhibitor, while clopdogrel was almost exclusively used in the other 9 trials. Number of treated lesions and total stent length were comparable across the trials (Table 1).

**Table 1:** Characteristics of the studies.

Trials	DAPT	RESET	OPTIMIZE	PRODIGY	EXCELLENT	SECURITY	ITALIC/ITALIC+	ISAR-SAFE	DES LATE	ARCTIC-Interruption
**Study Initiative**	Public-private collaboration	Investigator	Investigator	Investigator	Investigator	Investigator	Investigator	Investigator	Investigator	Investigator
**Study Design**	Double blind, Placebo-controlled	Open Label	Open Label	Open Label	Open Label	Open Label	Open Label	Double blind, Placebo-controlled	Open Label	Open Label
**Duration of Short-DAPT (months)**	12	3	3	6	6	6	6	6	12	12
**Duration of Prolonged-DAPT (months)**	30	12	12	24	12	12	24	12	36	18–30
**N of Patients Enrolled**	25682	2148	3211	2697	1443	1404	1894	4005	5045	1286
**N of Patients Randomized**	9961	2117	3119	1970	1443	1399	1850	4000	5045	1259
**N of Patients Analyzed**	9961	2117	3119	1970	1443	1399	1822	4000	5045	1259
**Timing of Randomization**	12 months	Index PCI	Index PCI	1 months	Index PCI	Index PCI	6 months	6 months	12 months	12 months
**Age (years)**	62	62	62	68	63	65	62	67, 67	62	64
**Female Sex (%)**	25	36	37	23	35	23	20	19	31	20
**Diabetes Mellitus (%)**	31	29	35	24	38	31	37	24	28	33
**Hypertension (%)**	75	62	87	72	73	73	65	91	58	61
**Current Smoker (%)**	25 (+within past year)	24	18	24	27	22	52	15	28	24
**Stroke or TIA (%)**	3.3	NA	2.4	3.9	6.6	NA	2.8	NA	2.1	5.2
**Prior PCI (%)**	31	3.2	20	18	8.9	18	23	NA	12	41
**Prior CABG (%)**	12	0.4	7.7	11	1.2	5.5	5.8	7.6	NA	6.5
**Prior MI (%)**	22	1.7	35	27	5.1	21	15	25	3.9	30
**Indication for PCI**										
** ACS (%)**	42	55	32	74	52	32	23	40	61	NA
** STEMI (%)**	10	14 (AMI)	0	33	3.1	0	0.2	8.1	12	NA
** Non-STEMI (%)**	15	NA	5.4	23	48 (NSTEMI+UAP)	0	7.2	10.3	11	NA
** Unstable Angina (%)**	17	40	NA	19		32	16	21.7	38	NA
**Region (Majority)**	North America	Asia	South America	Europe	Asia	Europe	Europe, Middle East	Europe	Asia	Europe
**Thienopyridine Drug**										
** Clopidogrel (%)**	65	100	100	100	100	99	99	NA	100	90
** Prasugrel (%)**	35	0	0	0	0	0.2	1.7	NA	0	8.5
**Type of Stents**										
**Second-generation DES (%)**	60	NA	100	50	75	96	100	71	NA	63
** EES (%)**	47	15	0	25	75	20	100	48	11 (vessel)	NA
** ZES Endeavor (%)**	13	50	100	25	0	42	NA	4.0	19 (vessel)	NA
** ZES Resolute (%)**	NA	21	0	0	0	NA	NA	11.4	NA	NA
** BES (%)**	NA	0	0	0	0	34	NA	8.4	NA	NA
**First-generation DES (%)**	38	NA	0	25	25	NA	NA	27	NA	41
** SES (%)**	11	14	0	0	25	NA	NA	25	44 (vessel)	NA
** PES (%)**	27	0	0	25	0	NA	NA	2.3	20 (vessel)	NA
**BMS (%)**	NA	0	0	25	0	1.2	NA	0.3	NA	NA
**N of Treated Lesions**	1.3	1.3	1.3	1.5	1.3	1.4	NA	NA	1.4 (vessel)	NA
**N of Stents Used**	1.5	NA	1.6	1.86	1.6	1.6	1.7	1.68	1.3 (lesion)	NA
**Total Stent Length (mm)**	28	30	43	30, 30 (median)	36	31	38	28, 28 (median)	38	NA
**Treated Vessel**										
** LMCA (%)**	0.8	0	1.3	5.6	0	0	1.2	0.3	2.8	3.3
** LAD (%)**	41	53	47	53	62	44	73	40	50	53
** RCA (%)**	32	27	28	36	NA	22	53	33	27	33
** CX (%)**	23	20	24	32	NA	14	49	25	19	31
** Graft (%)**	3	NA	NA	NA	NA	NA	5.4	1.4	0.1	1

DAPT = dual antiplatelet therapy, N = number, NA = not available, TIA = transient ischemia attack, PCI = percutaneous coronary intervention, CABG = coronary artery bypass grafting, MI = myocardial infarction, ACS = acute coronary syndrome, STEMI = ST-segment elevation myocardial infarction, CAD = coronary artery disease, DES = drug-eluting stent, EES = everolimus-eluting stent, ZES = zotarolimus-eluting stent, BES = biolimus-eluting stent, SES = sirolimus-eluting stent, PES = paclitaxel-eluting stent, LMCA = left main coronary artery, LAD = left anterior descending artery, RCA = right coronary artery, and CX = circumflex artery.

### Definition of endpoints among included trials

All the trials adopted the composite endpoints as the primary endpoint. However, the components of the composite endpoints were variable across studies. Net clinical benefit including bleeding events was evaluated as the primary endpoint in 5 studies. The definitions of bleeding were also variable across studies (Table 2).

**Table 2:** Definitions of the study endpoint.

Trials	DAPT	RESET	OPTIMIZE	PRODIGY	EXCELLENT	SECURITY	ITALIC /ITALIC+	ISAR-SAFE	DES LATE	ARCTIC -Interruption
**Primary Composite Endpoint**	Death	Cardiac Death	Death	Death	Cardiac Death	Cardiac Death	Death	Death	Cardiac Death	Death
	MI	MI	MI	Non-fatal MI	MI	MI	MI	MI	MI	MI
	Stroke	ST	Stroke	Stroke	TVR	Stroke	Stroke	ST	Stroke	ST
		Ischemia-driven TVR	Major Bleeding			ST	Emergency TVR	Stroke		Stroke
		Bleeding				Bleeding	Bleeding	Bleeding		Urgent Coronary Revascularization
**MI**	ARC	ARC	WHO	UD	ARC	Original*	ARC	TIMI	UD	ARC
**ST**	ARC (Definite/Probable)	ARC (Definite/Probable)	ARC (Definite/Probable)	ARC (Definite/Probable)	ARC (Definite/Probable)	ARC (Definite/Probable)	ARC (No Detail)	ARC (Definite/Probable)	ARC Definite	ARC (All-definite)
**Stroke**	Ischemic/Hemorrhagic	-	CVA	CVA (+TIA)	CVA	Ischemic/Hemorrhagic	Hemorrhagic/Non-hemorrhagic	Ischemic/Hemorrhagic	Ischemic/Hemorrhagic	Stroke or TIA
**Bleeding**	GUSTO Severe/Moderate	TIMI Major	Major REPLACE-2 and GUSTO Severe	BARC 2, 3, 5	TIMI Major	BARC 3 or 5	TIMI Major	TIMI Major	TIMI Major	STEEPLE

* Definition of MI for SECURITY: Spontaneous MI was defined by the following criteria: cardiac enzyme elevation (troponin T/I or creatine kinase-myocardial band) above the upper normal limit associated with at least 1 ischemic symptom; development of Q waves on the electrocardiogram; electrocardiogram changes indicative of ischemia or coronary artery intervention.

MI = myocardial infarction, ST = stent thrombosis, TVR = target-vessel revascularization, TIA = transient ischemic attack, TLR = target lesion revascularization, CABG = coronary Artery bypass grafting, TIMI = Thrombolysis in myocardial infarction, UD = Universal Definition of Myocardial Infarction, ARC = Academic Research Consortium, WHO = historical extended WHO definition, CVA = Cerebrovascular Accident, REPLACE-2 = Modified Randomized Evaluation in PCI Linking Angiomax to Reduced Clinical Events II, GUSTO = The Global Use of Strategies to Open Occluded Arteries definition, and STEEPLE = Safety and Efficacy of Enoxaparin in PCI Patients, an International Randomized Evaluation.

In all the trials, ST was classified by the Academic Research Consortium (ARC) definition. MI was variably defined by the ARC definition in 5 trials, the universal definition in 2 trials, the world health organization (WHO) definition in 1 trial, Thrombolysis in Myocardial Infarction (TIMI) study group definition in 1 trial, and original definition in 1 trial.

### Effect of prolonged-DAPT on clinical outcomes: Comparison between DAPT trial and other trials

**Fig 1:**
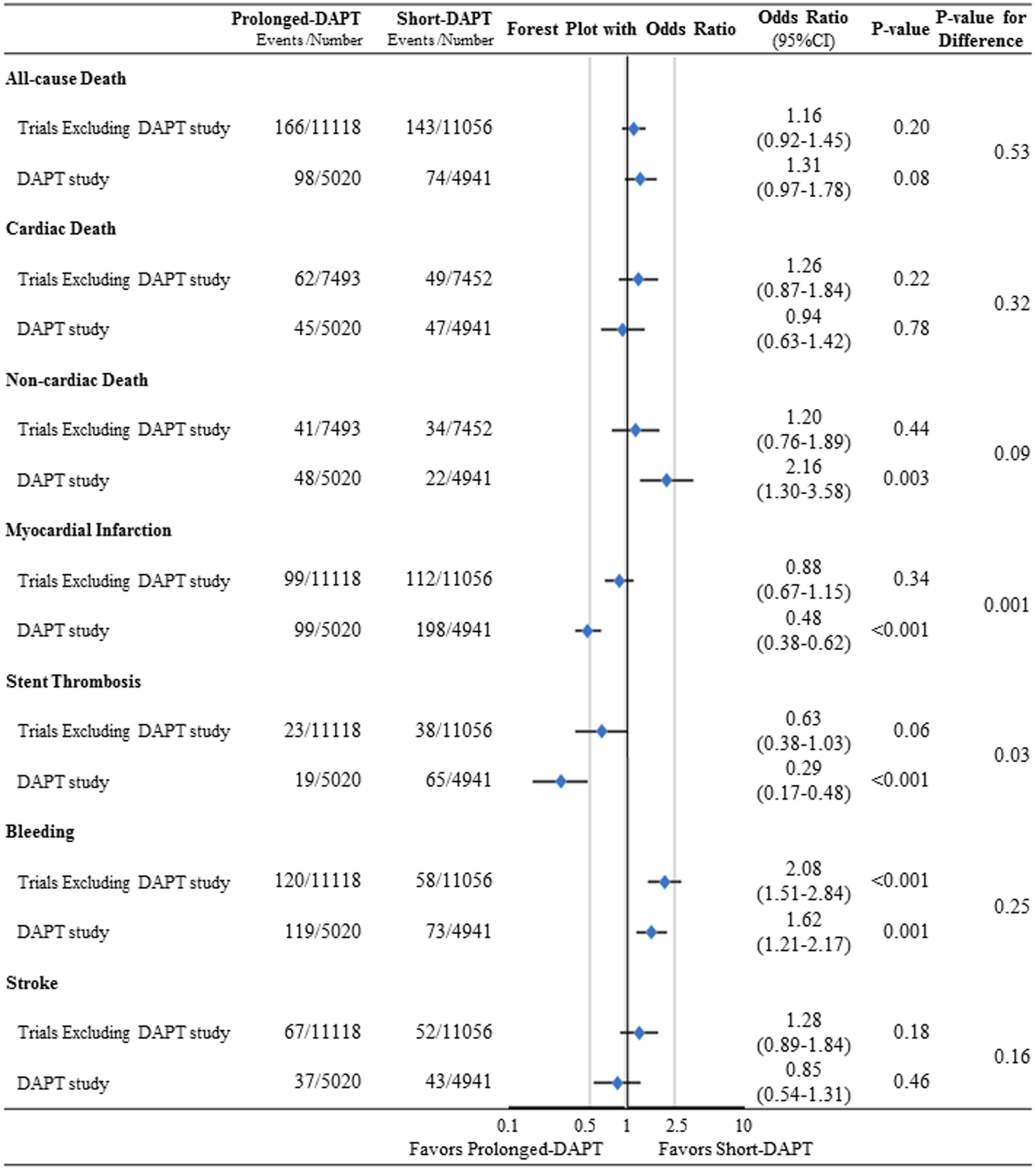
Forest plot with OR of prolonged-DAPT relative to short-DAPT for each clinical outcome for the DAPT trial and the pooled population excluding the DAPT trial. ORs are shown on a logarithmic scale. CI = confidence interval, DAPT = dual anti-platelet therapy, and OR = odds ratio.

The results from the DAPT study (N = 9961) were different from the pooled results of the 9 other trials (N = 22174). Details of the pooled analysis of the 9 other trials including the forest plots with OR of prolonged-DAPT relative to short-DAPT for each clinical outcomes and the Cochrane Q test and I-square statistics were shown in Supplemental Figures (S2–S6 Figs). There was no evidence of significant publication bias among 9 trials (S7 Fig). In the DAPT study, prolonged-DAPT as compared with short-DAPT was associated with marked risk reduction for ST and MI, while in the other trials, there was a trend favoring prolonged-DAPT over short-DAPT in terms of ST and MI, but the extent of risk reduction was much smaller than that in the DAPT study (ST: OR 0.29 [95%CI 0.17–0.48] versus pooled OR 0.63 [95%CI 0.38–1.03]: P = 0.03 for difference, and MI: OR 0.48 [95%CI 0.38–0.62] versus pooled OR 0.88 [95%CI 0.67–1.15]: P = 0.001 for difference) (Fig 1). On the other hands, the trends for excess risk of prolonged-DAPT relative to short-DAPT for all-cause death (OR 1.31 [95%CI 0.97–1.78] versus pooled OR 1.16 [95%CI 0.92–1.45]: P = 0.53 for difference), and bleeding (OR 1.62 [95%CI 1.21–2.17] versus pooled OR 2.08 [95%CI 1.51–2.84]: P = 0.25 for difference) were consistently seen in both the DAPT and other trials (Fig 1). However, the excess non-cardiac mortality risk with prolonged DAPT in the DAPT study was not clearly seen in the other trials (OR 2.16 [95%CI 1.3–3.58] versus pooled OR 1.20 [95%CI 0.76–1.89]: P = 0.09 for difference) (Fig 1).

### Annual rates of endpoint events during aspirin monotherapy: Comparison between DAPT trial and other trials

**Fig 2:**
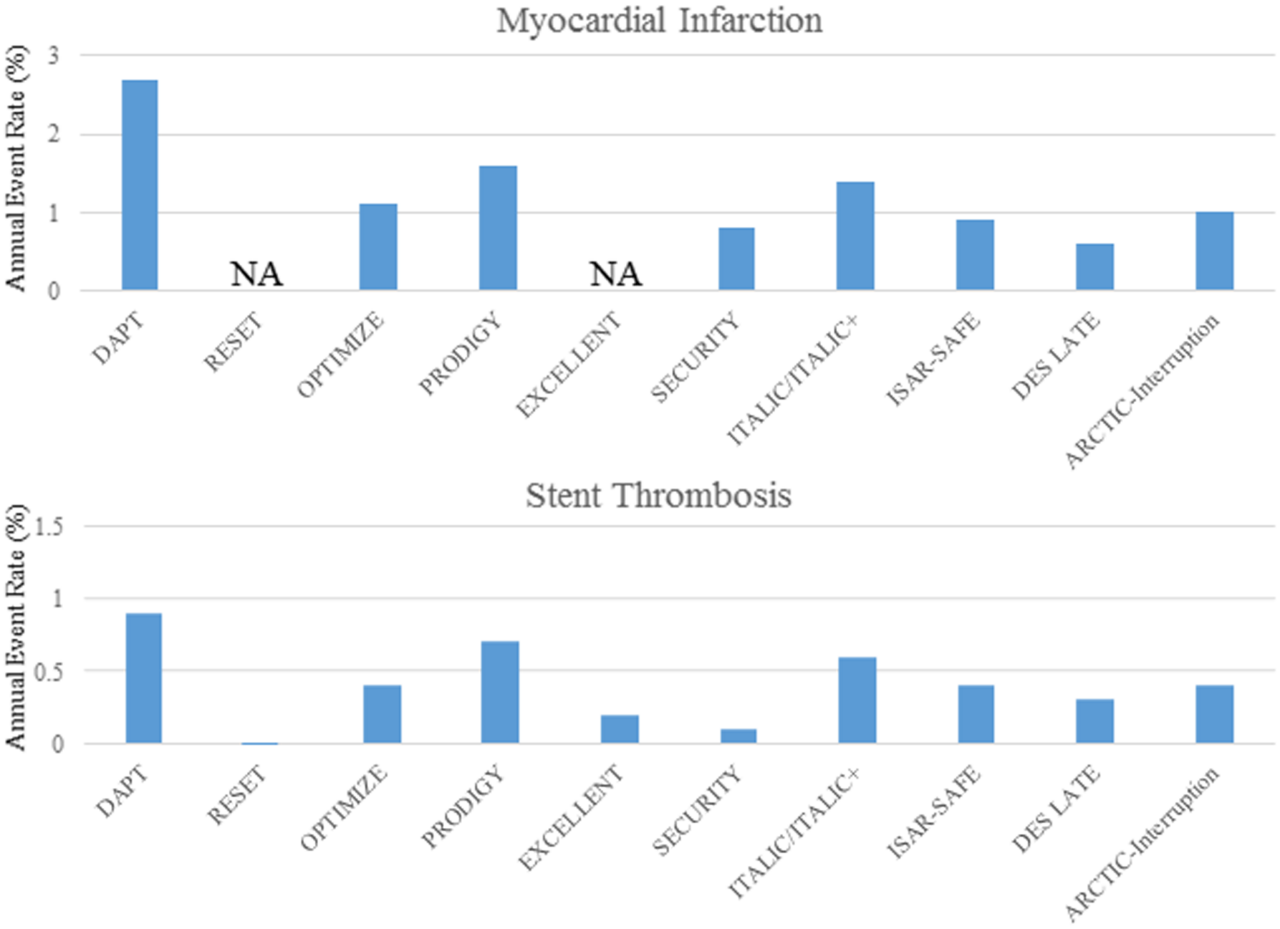
Estimated annual event rates for myocardial infarction and stent thrombosis in the short-DAPT group during aspirin mono-therapy. The landmark data was not available for myocardial infarction in the RESET and EXCELLENT trials. NA = not available.

The annual rates of MI and ST during aspirin mono-therapy were higher in the DAPT study than in the other trials (2.7% versus 0.6–1.6%, and 0.9% versus 0–0.7%, respectively) (Table 3, and Fig 2). The annual rate of major bleeding was also higher in the DAPT study than in the other studies. The corresponding rates for all-cause death, and stroke were comparable between the DAPT study and the other trials (Table 3).

**Table 3:** Annual event rate on each randomized regimen.

Trials		DAPT	RESET	OPTIMIZE	PRODIGY	EXCELLENT	SECURITY	ITALIC/ITALIC+	ISAR-SAFE	DES LATE	ARCTIC-Interruption
**Death**	Short DAPT	1	NA	2.5	NA	NA	0.7	1.8	0.5	0.7	1
	Prolonged DAPT	1.3	NA	2.3	NA	NA	1	1.6	0.8	1	0.8
**Myocardial Infarction**	Short DAPT	2.7	NA	1.1	1.6	NA	0.8	1.4	0.9	0.6	1
	Prolonged DAPT	1.4	NA	0.8	1.8	NA	0.7	0.8	0.9	0.4	1
**ST (definite/probable)**	Short DAPT	0.9	0	0.4	0.7	0.2	0.1	0.6	0.4	0.3	0.4
	Prolonged DAPT	0.3	0.3	0.1	0.6	0	0	0	0.3	0.2	0
**Major Bleeding**	Short DAPT	1.1	NA	0.3	NA	NA	0.2	0	0.3	0.6	0.1
	Prolonged DAPT	1.7	NA	0.5	NA	NA	0.2	0.6	0.4	0.7	0.8
**Stroke**	Short DAPT	0.6	NA	0.4	0.3	NA	0.3	0	0.5	0.5	0.4
	Prolonged DAPT	0.5	NA	0.1	1.1	NA	0.1	0.8	0.4	0.5	0.6

Data were presented by percent per year.

DAPT = Dual Anti-platelet Therapy, and NA = Not Available.

## Discussion

The main findings of the current analysis were the following; (1) The results from the DAPT study were different from the pooled results of the 9 other trials in terms of the effect of prolonged-DAPT on MI, and definite or probable ST; (2) The annual rates of MI and ST during aspirin mono-therapy were higher in the DAPT study than in the other trials.

The recently reported DAPT study, the largest trial evaluating the optimal duration of DAPT after coronary stent implantation, demonstrated that prolonged-DAPT up to 30-month as compared with short-DAPT up to 12-month was associated with marked risk reduction for MI and ST, leading to significant risk reduction for the primary composite endpoint. However, in the 9 trials other than DAPT study enrolling >20000 patients, prolonged-DAPT for 12- to 48-month as compared with short-DAPT for 3- to 12-month was not associated with significant risk reduction for MI and ST. One of the reasons for the different results of the DAPT study and the other studies might be that the DAPT study is heavily powered to detect differences in ST and MI while the other studies were only powered to detect differences in the composite endpoints. However, the remarkable difference between the DAPT study and the other trials was the higher annual rate of MI during aspirin mono-therapy in the DAPT study as compared with those in the other trials, although we should be cautious in comparing the event rates across different studies. The cardiovascular mortality was also similar between the short- and prolonged-DAPT groups despite a large risk reduction for MI by prolonging DAPT in the largest DAPT study, although this might be consistent with the contemporary studies evaluating the effect of MI on cardiovascular mortality due to the improved interventional and pharmacologic management of MI.

The primary endpoints of the DAPT study were ST and a composite of death, MI, or stroke. There was a signal suggesting increased mortality with prolonged DAPT in the DAPT study and in the other 9 trials as well as in the recently published meta-analyses.[7–13] The positive result of the DAPT study for the one of the co-primary endpoint was exclusively related to marked reduction of MI. For the construction of the composite endpoint, we should assume that each component should be of comparable clinical importance.[14] However, there is a concern that a small MI defined by the slight increase of the cardiac biomarker criteria especially for troponins would not have clinical importance comparable to death.

It is still controversial after the DAPT study presentation whether DAPT should be extended beyond 1-year after coronary stent implantation. In the present study, we found consistent increase of all-cause death and bleeding with prolonged-DAPT in both the DAPT study and other trials. Furthermore, recent meta-analyses comparing short- versus prolonged-DAPT reported a borderline increase of all-cause death, and highly significant increase of non-cardiac death with prolonged-DAPT.[9–11] However, the DAPT study clearly demonstrated that prolonged-DAPT substantially reduced ischemic events, particularly ST, beyond 1-year after coronary stent implantation. Also, the United States Food and Drug Administration released updated safety communication stating that the DAPT with clopidogrel does not change the risk of death, using the meta-analysis of DAPT studies targeting large spectrum of disease.[15] Therefore, balancing the risks and benefits of DAPT is needed in considering the antithrombotic therapy for patients after coronary stenting. In the future it will be important to clarify the patients who derive the most benefit from prolonged DAPT, by analyzing patients stratified into several clinical situations; acute coronary syndromes, prior history of bleeding/cancer, left main disease/bifurcation lesions, stent characteristics, and so on. Prolonged-DAPT could be an option in patients with high ischemic risk, but without high bleeding risk.

### Limitations

We did not conduct a formal meta-analysis including all the searched trials, because the result of the largest DAPT study was so different from those of the other 9 trials. We considered that clarifying the differences and similarities between the DAPT study and other trials would be more important than drawing a conclusion by simply pooling the results from all the trials. There are some limitations in the current report. First and most importantly, it is challenging to compare the results of different trials. However, considering the huge clinical impact of the DAPT study result, we should address the reasons for the difference between the DAPT study and the other trials. It is certain that the annual rate of MI on aspirin mono-therapy in the DAPT study was much higher than those in other trials. However, we do not know the details of MI events in the DAPT study. Therefore, it remains speculative to argue that a large proportion of MI events reported in the DAPT study could be small MI that were not regarded as the endpoint events in the investigator-driven trials. Second, another meta-analysis comparing short- versus prolonged-DAPT that also included non-PCI trials did not suggest increased mortality with prolonged-DAPT.[16] Also, in the present study, the excess non-cardiac mortality risk with prolonged-DAPT in the DAPT study was not clearly seen in the other trials. Therefore, the finding within the DAPT study mortality signal could be a chance finding. Third, only 3 of the 9 trials included in the meta-analysis evaluated DAPT beyond 12-month after coronary stent implantation. However, the risk for cardiovascular events including ST beyond 30-day after DES implantation seemed to be constant from several long-term studies of DES.[17,18] Forth, the timings of randomization were different across studies. In the DAPT study, 14034 patients out of 25682 enrolled patients were not randomized at 12-month, suggesting inclusion of those patients with relatively low risk for bleeding. However, it would be difficult to explain the favorable ischemic outcome in the DAPT study by the inclusion of patients with low bleeding risk, because significantly higher risk for bleeding was also seen in the DAPT study as in the other trials. Finally, the DAPT study is the only study among the listed trials in which patients were primarily enrolled within North America. The observed differences in the event rates and the effects of prolonged-DAPT relative to short DAPT between the DAPT study and other trials might be related to the differences in the practice pattern, insurance systems, races, and the way to execute clinical trials between North America and other geographic areas.

## Conclusions

Given the difference between the DAPT study and other trials, future studies should focus on certain subgroups of patients that are more or less likely to benefit from longer duration DAPT.

## Supporting information

S1 Fig Study selection flow chart according to the PRISMA (Preferred Reporting Items for Systematic reviews and Meta-Analyses) statement. (TIF)

S2 Fig Forest plot with OR of prolonged-DAPT relative to short-DAPT for all-cause death for the pooled population excluding the DAPT trial. (TIF)

S3 Fig Forest plot with OR of prolonged-DAPT relative to short-DAPT for cardiac death for the pooled population excluding the DAPT trial. (TIF)

S4 Fig Forest plot with OR of prolonged-DAPT relative to short-DAPT for non-cardiac death for the pooled population excluding the DAPT trial. (TIF)

S5 Fig Forest plot with OR of prolonged-DAPT relative to short-DAPT for myocardial infarction for the pooled population excluding the DAPT trial. (TIF)

S6 Fig Forest plot with OR of prolonged-DAPT relative to short-DAPT for stent thrombosis for the pooled population excluding the DAPT trial. (TIF)

S7 Fig Forest plot with OR of prolonged-DAPT relative to short-DAPT for bleeding for the pooled population excluding the DAPT trial. (TIF)

S8 Fig Forest plot with OR of prolonged-DAPT relative to short-DAPT for stroke for the pooled population excluding the DAPT trial. (TIF)

S9 Fig Begg’s funnel plot and Egger’s test for evaluation of publication bias (A: all-cause death, B: cardiac death, C: non-cardiac death, D: myocardial infarction, E: stent thrombosis, F: bleeding, and G: stroke). (TIF)

S1 Table List of the 10 trials analyzed in the current study. (DOCX)

S2 Table Cochrane’s collaboration tool for assessing risk of bias among included studies. (DOCX)

S3 Table PRISMA check list for the pooled analysis of the 9 trials. (DOCX)

## References

[1] Levine GN, Bates ER, Blankenship JC, Bailey SR, Bittl JA, Cercek B, et al (2011) 2011 ACCF/AHA/SCAI Guideline for Percutaneous Coronary Intervention: executive summary: a report of the American College of Cardiology Foundation/American Heart Association Task Force on Practice Guidelines and the Society for Cardiovascular Angiography and Interventions. Circulation 124: 2574–2609. doi: 10.1161/CIR.0b013e31823a5596 22064598 10.1161/CIR.0b013e31823a5596

[2] Stefanini GG, Siontis GC, Cao D, Heg D, Juni P, Windecker S, et al (2014) Short versus long duration of DAPT after DES implantation: a meta-analysis. J Am Coll Cardiol 64: 953–954. doi: 10.1016/j.jacc.2014.06.1158 25169183 10.1016/j.jacc.2014.06.1158

[3] Authors/Task Force members, Windecker S, Kolh P, Alfonso F, Collet JP, Cremer J, et al (2014) 2014 ESC/EACTS Guidelines on myocardial revascularization: The Task Force on Myocardial Revascularization of the European Society of Cardiology (ESC) and the European Association for Cardio-Thoracic Surgery (EACTS)Developed with the special contribution of the European Association of Percutaneous Cardiovascular Interventions (EAPCI). Eur Heart J 35: 2541–2619. doi: 10.1093/eurheartj/ehu278 25173339 10.1093/eurheartj/ehu278

[4] Mauri L, Kereiakes DJ, Yeh RW, Driscoll-Shempp P, Cutlip DE, Steg PG, et al (2014) Twelve or 30 Months of Dual Antiplatelet Therapy after Drug-Eluting Stents. N Engl J Med 371: 2155–2166. doi: 10.1056/NEJMoa1409312 25399658 10.1056/NEJMoa1409312 PMC4481318

[5] Higgins J, Green S, (editors). Cochrane Handbook for Systematic Reviews of Interventions Version 5.1.0 [updated March 2011]. The Cochrane Collaboration, 2011. www.cochrane-handbook.org.

[6] Higgins JP, Thompson SG, Deeks JJ, Altman DG (2003) Measuring inconsistency in meta-analyses. BMJ 327: 557–560. doi: 10.1136/bmj.327.7414.557 12958120 10.1136/bmj.327.7414.557 PMC192859

[7] Abo-Salem E, Alsidawi S, Jamali H, Effat M, Helmy T (2015) Optimal duration of dual antiplatelet therapy after drug eluting stents: Meta-analysis of randomized trials. Cardiovasc Ther 33: 253–263. doi: 10.1111/1755-5922.12137 26010419 10.1111/1755-5922.12137

[8] Giustino G, Baber U, Sartori S, Mehran R, Mastoris I, Kim AS et al (2015) Duration of dual antiplatelet therapy after drug-eluting stent implantation: a systematic review and meta-analysis of randomized controlled trials. J Am Coll Cardiol 65: 1298–1310. doi: 10.1016/j.jacc.2015.01.039 25681754 10.1016/j.jacc.2015.01.039

[9] Cassese S, Byrne RA, Ndrepepa G, Schunkert H, Fusaro M, Kastrati A, et al (2015) Prolonged dual antiplatelet therapy after drug-eluting stenting: meta-analysis of randomized trials. Clin Res Cardiol 104: 887–901. doi: 10.1007/s00392-015-0860-1 25903112 10.1007/s00392-015-0860-1

[10] Navarese EP, Andreotti F, Schulze V, Kolodziejczak M, Buffon A, Brouwer M, et al (2015) Optimal duration of dual antiplatelet therapy after percutaneous coronary intervention with drug eluting stents: meta-analysis of randomised controlled trials. BMJ 350: h1618 [Epub ahead of print] doi: 10.1136/bmj.h1618 25883067 10.1136/bmj.h1618 PMC4410620

[11] Palmerini T, Benedetto U, Bacchi-Reggiani L, Della Riva D, Biondi-Zoccai G, Feres F, et al (2015) Mortality in patients treated with extended duration dual antiplatelet therapy after drug-eluting stent implantation: a pairwise and Bayesian network meta-analysis of randomised trials. Lancet 385: 2371–2382. doi: 10.1016/S0140-6736(15)60263-X 25777667 10.1016/S0140-6736(15)60263-X

[12] Spencer FA, Prasad M, Vandvik PO, Chetan D, Zhou Q, Guyatt G, et al (2015) Longer Versus Shorter Duration Dual-Antiplatelet Therapy After Drug-Eluting Stent Placement: A Systematic Review and Meta-analysis. Ann Intern Med 163: 118–126. doi: 10.7326/M15-0083 26005909 10.7326/M15-0083

[13] Verdoia M, Schaffer A, Barbieri L, Montalescot G, Collet JP, Colombo A, et al (2015) Optimal Duration of Dual Antiplatelet Therapy After DES Implantation: A Meta-Analysis of 11 Randomized Trials. Angiology 67: 224–238. doi: 10.1177/0003319715586500 26069031 10.1177/0003319715586500

[14] Kaul S, Diamond GA (2010) Trial and error. How to avoid commonly encountered limitations of published clinical trials. J Am Coll Cardiol 55: 415–427. 20117454 10.1016/j.jacc.2009.06.065

[15] U.S. Food and Drug Administration (2015) FDA Drug Safety Communication: FDA review finds long-term treatment with blood-thinning medicine Plavix (clopidogrel) does not change risk of death. http://wwwfdagov/Drugs/DrugSafety/ucm471286htm, accessed at November 5, 2016.

[16] Elmariah S, Mauri L, Doros G, Galper BZ, O'Neill KE, Steg PG, et al (2014) Extended duration dual antiplatelet therapy and mortality: a systematic review and meta-analysis. The Lancet 28: 792–798. 10.1016/S0140-6736(14)62052-3 PMC4386690 25467565

[17] Natsuaki M, Morimoto T, Furukawa Y, Nakagawa Y, Kadota K, Yamaji K, et al (2014) Late adverse events after implantation of sirolimus-eluting stent and bare-metal stent: long-term (5–7 years) follow-up of the Coronary Revascularization Demonstrating Outcome study-Kyoto registry Cohort-2. Circ Cardiovasc Interv 7: 168–179. doi: 10.1161/CIRCINTERVENTIONS.113.000987 24550439 10.1161/CIRCINTERVENTIONS.113.000987

[18] Tada T, Byrne RA, Simunovic I, King LA, Cassese S, Joner M, et al (2013) Risk of stent thrombosis among bare-metal stents, first-generation drug-eluting stents, and second-generation drug-eluting stents: results from a registry of 18,334 patients. JACC Cardiovasc Interv 6: 1267–1274. doi: 10.1016/j.jcin.2013.06.015 24355117 10.1016/j.jcin.2013.06.015

