# Supplementary figures and images for: Short versus prolonged dual antiplatelet therapy (DAPT) duration after coronary stent implantation: A comparison between the DAPT study and 9 other trials evaluating DAPT duration
Source: PLoS One. 2017 Sep 20;12(9):e0174502. doi: 10.1371/journal.pone.0174502 (PMC5607128; doi:10.1371/journal.pone.0174502)

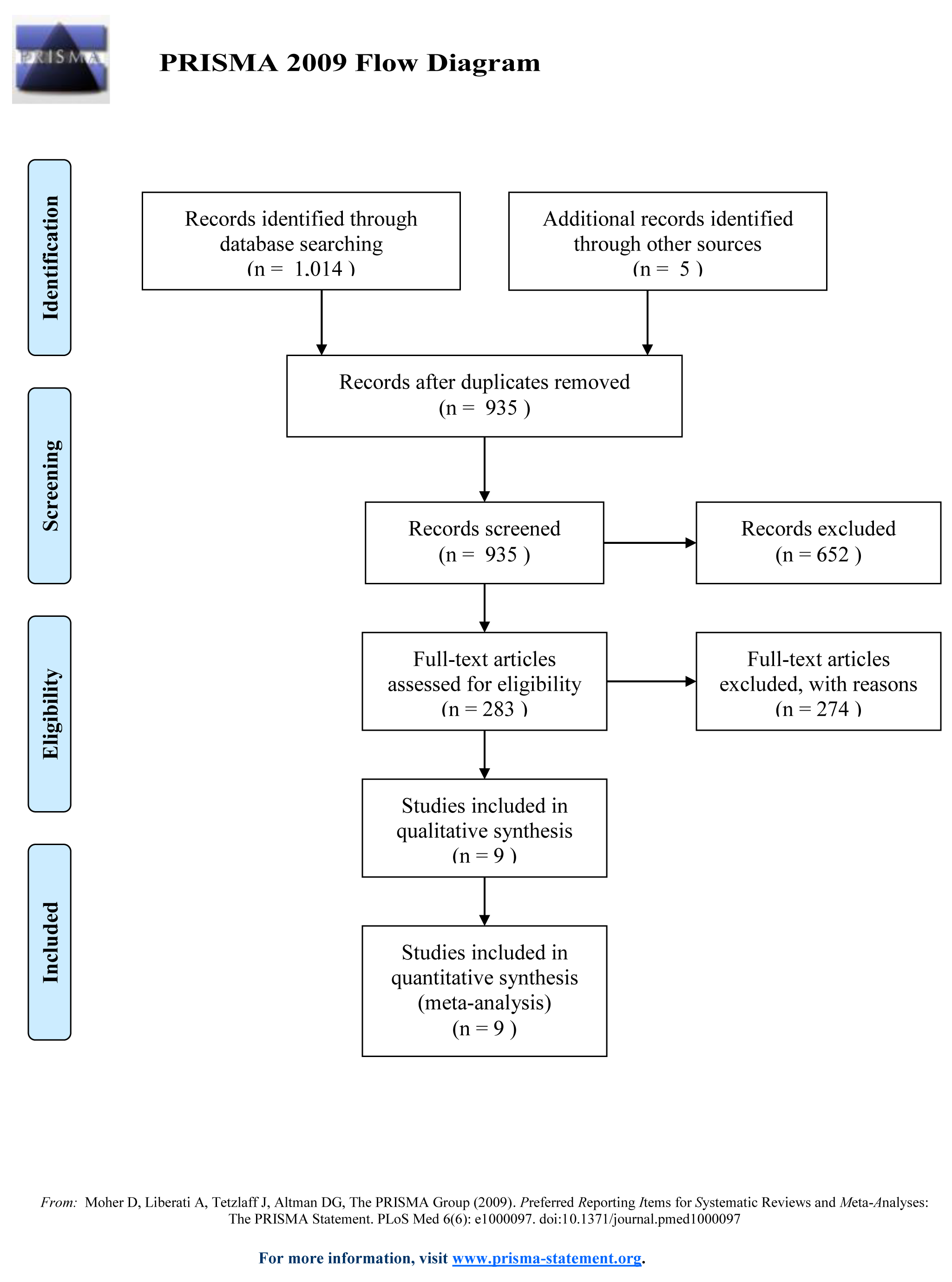

Supplement: S1 Fig — (TIF) [file pone.0174502.s001.tif]

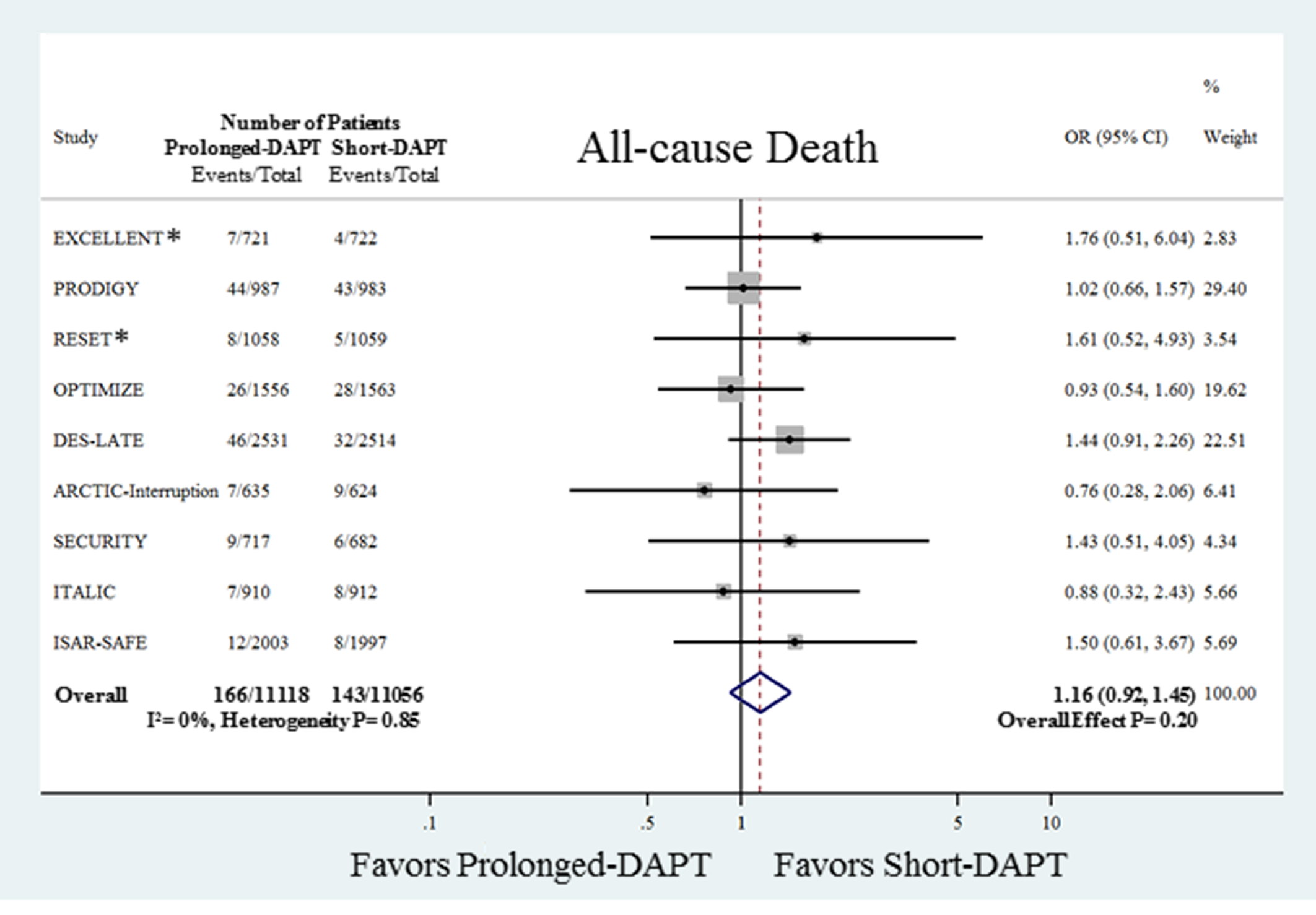

Supplement: S2 Fig — (TIF) [file pone.0174502.s002.tif]

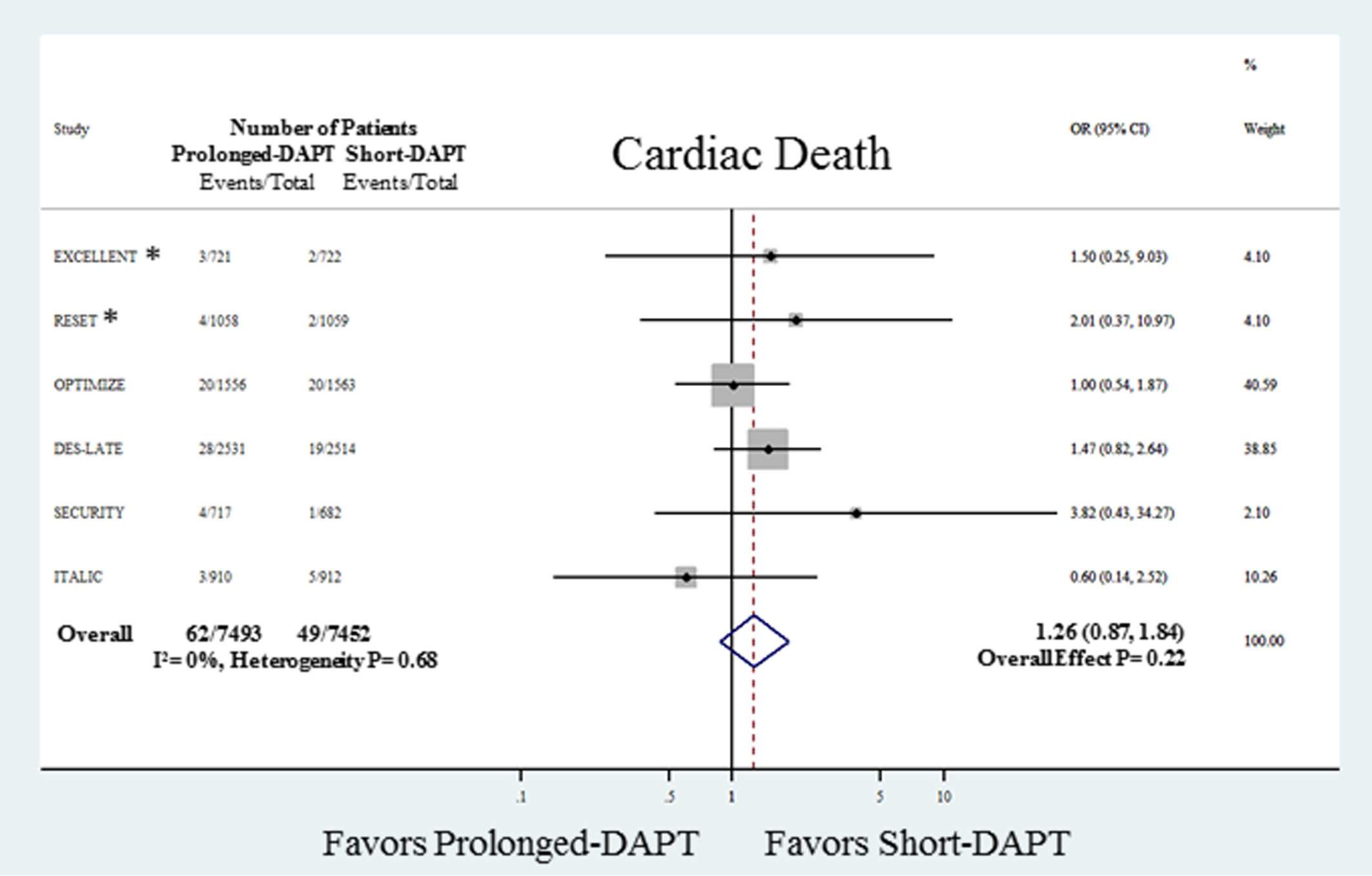

Supplement: S3 Fig — (TIF) [file pone.0174502.s003.tif]

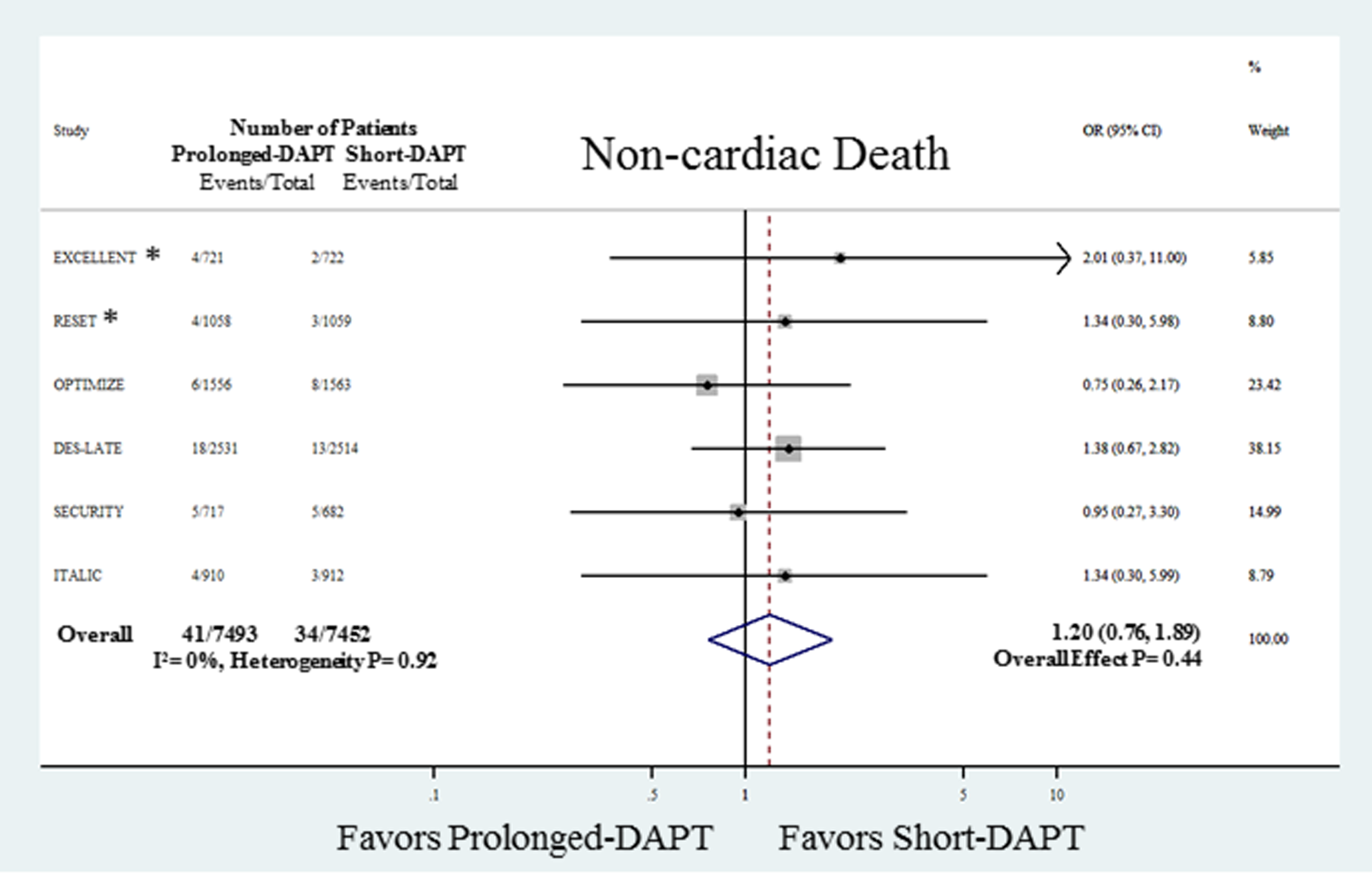

Supplement: S4 Fig — (TIF) [file pone.0174502.s004.tif]

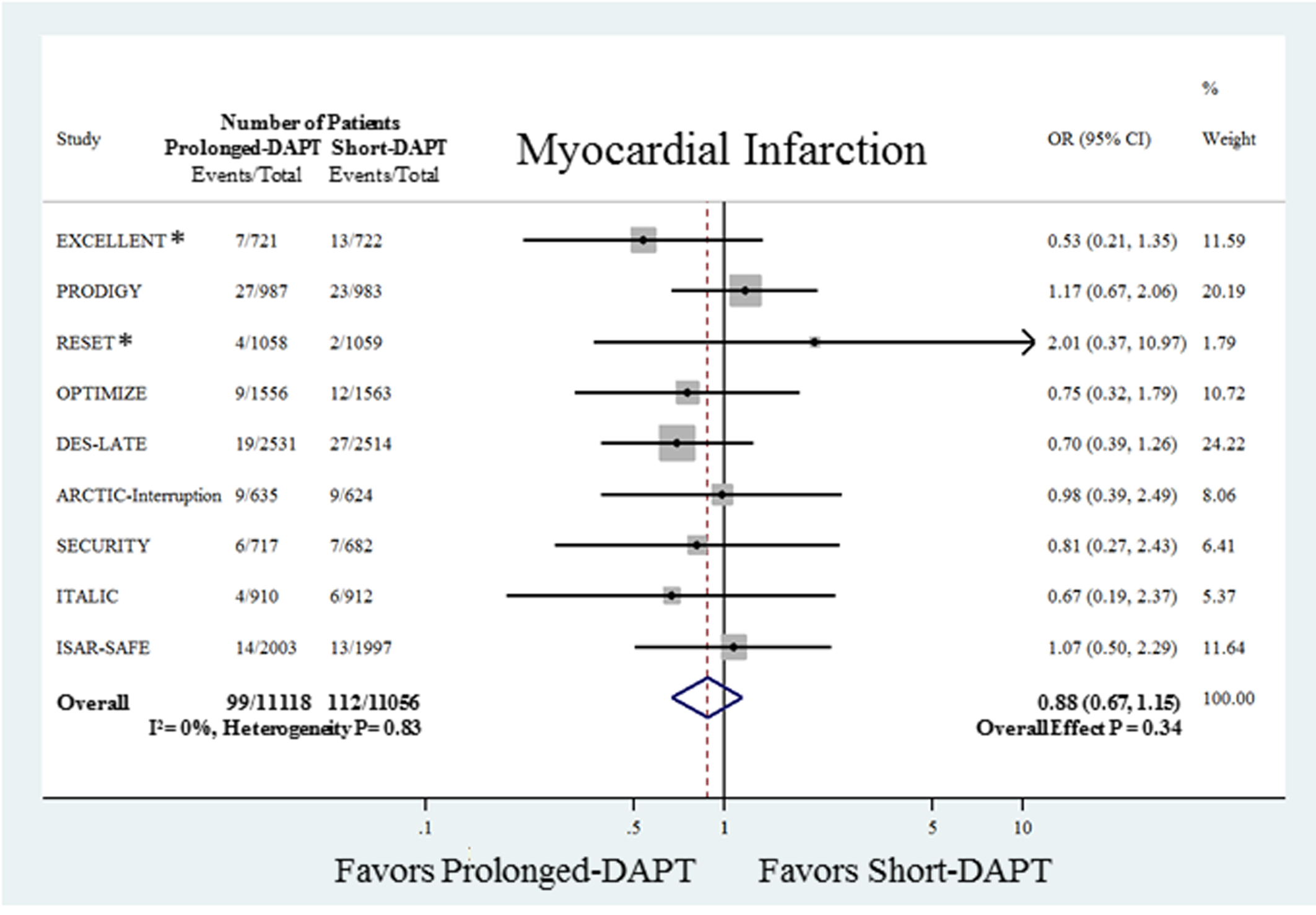

Supplement: S5 Fig — (TIF) [file pone.0174502.s005.tif]

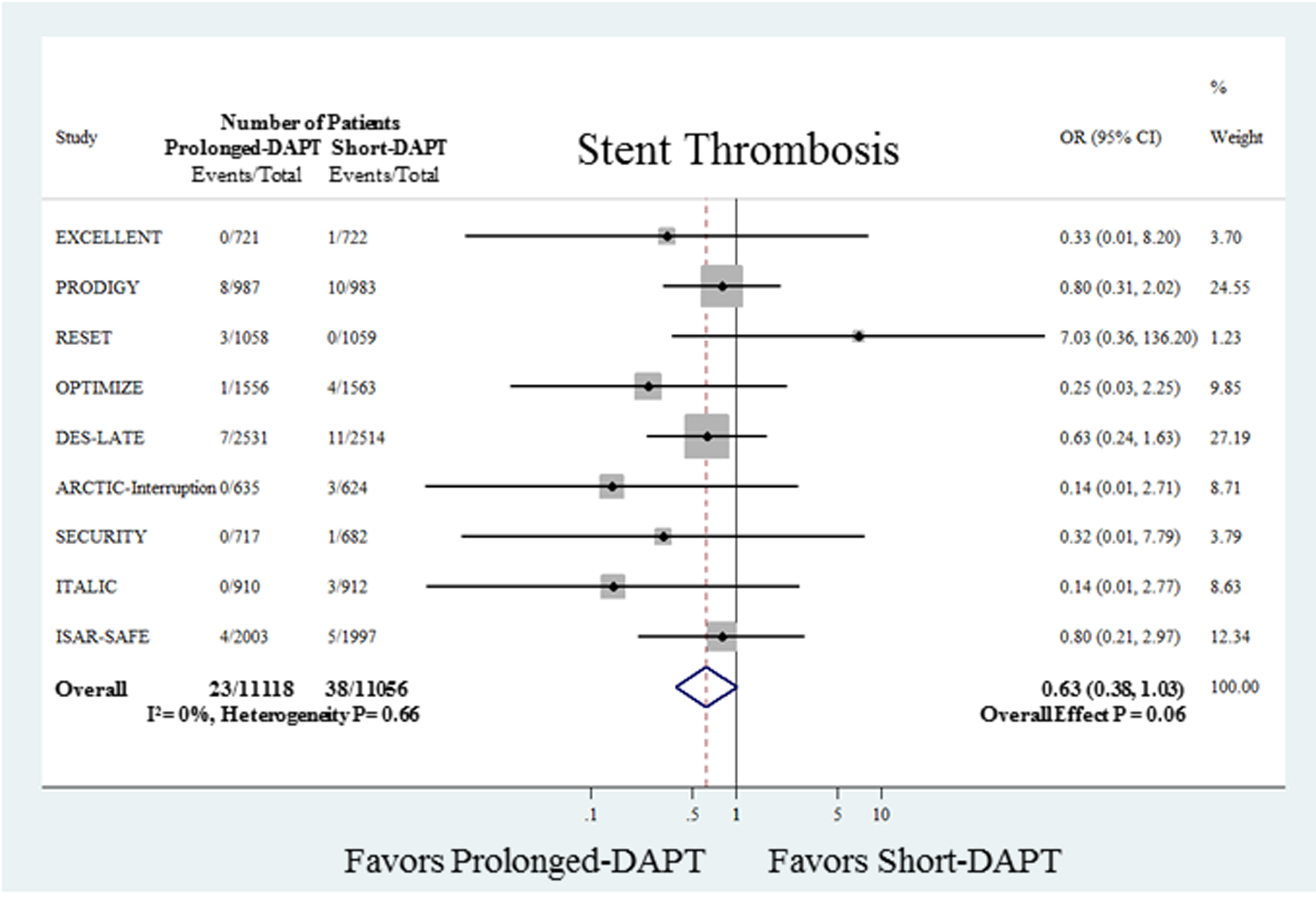

Supplement: S6 Fig — (TIF) [file pone.0174502.s006.tif]

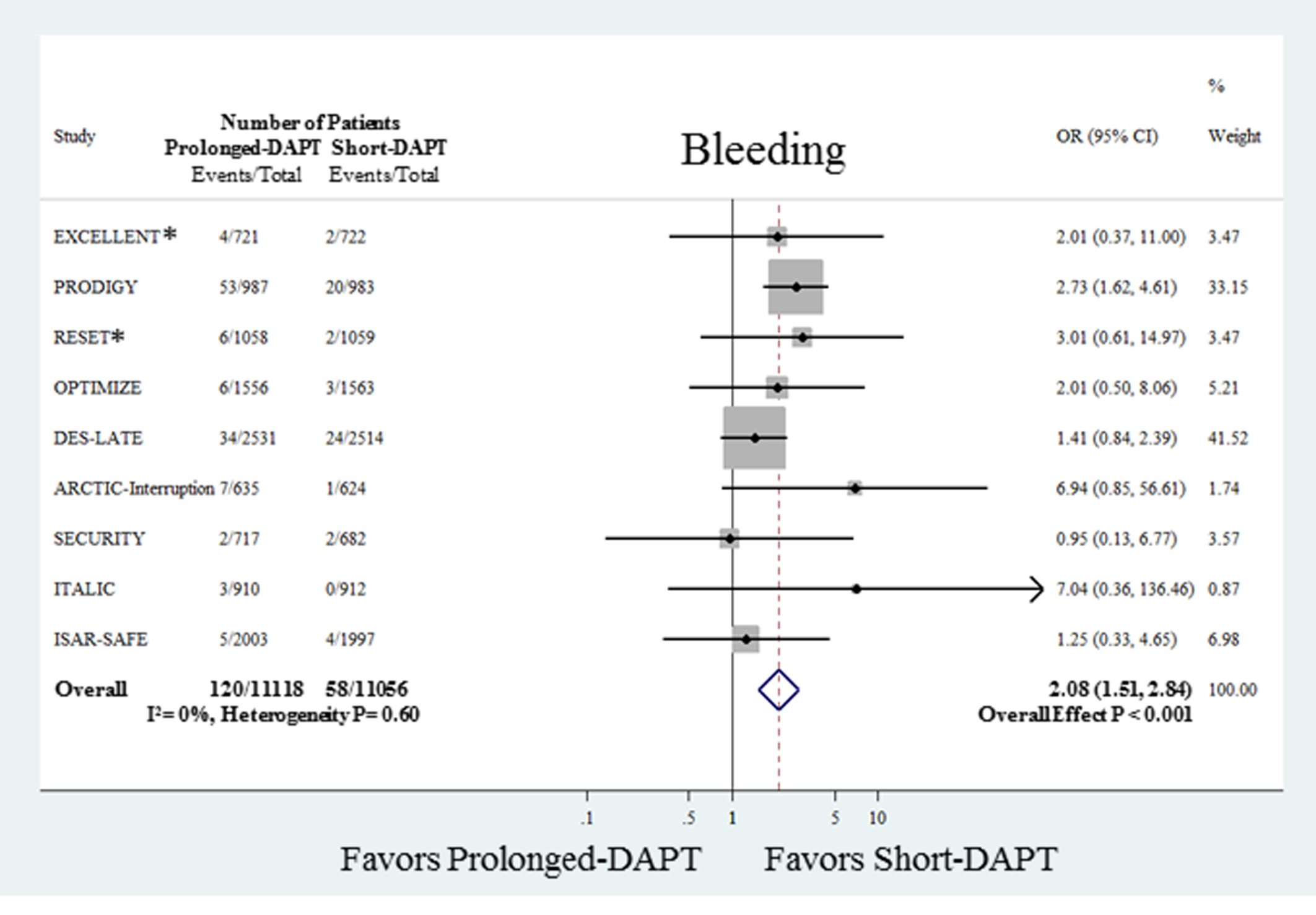

Supplement: S7 Fig — (TIF) [file pone.0174502.s007.tif]

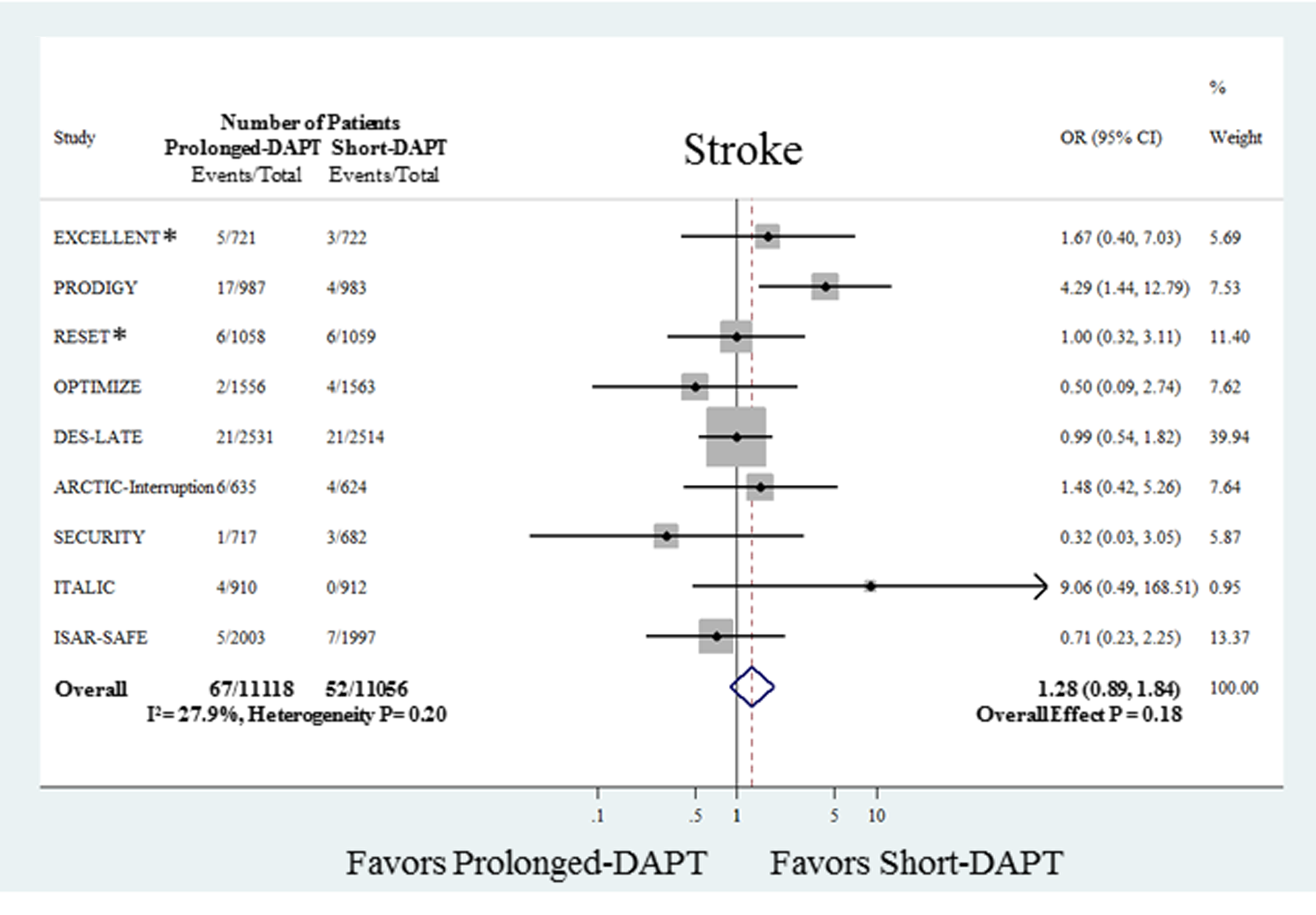

Supplement: S8 Fig — (TIF) [file pone.0174502.s008.tif]

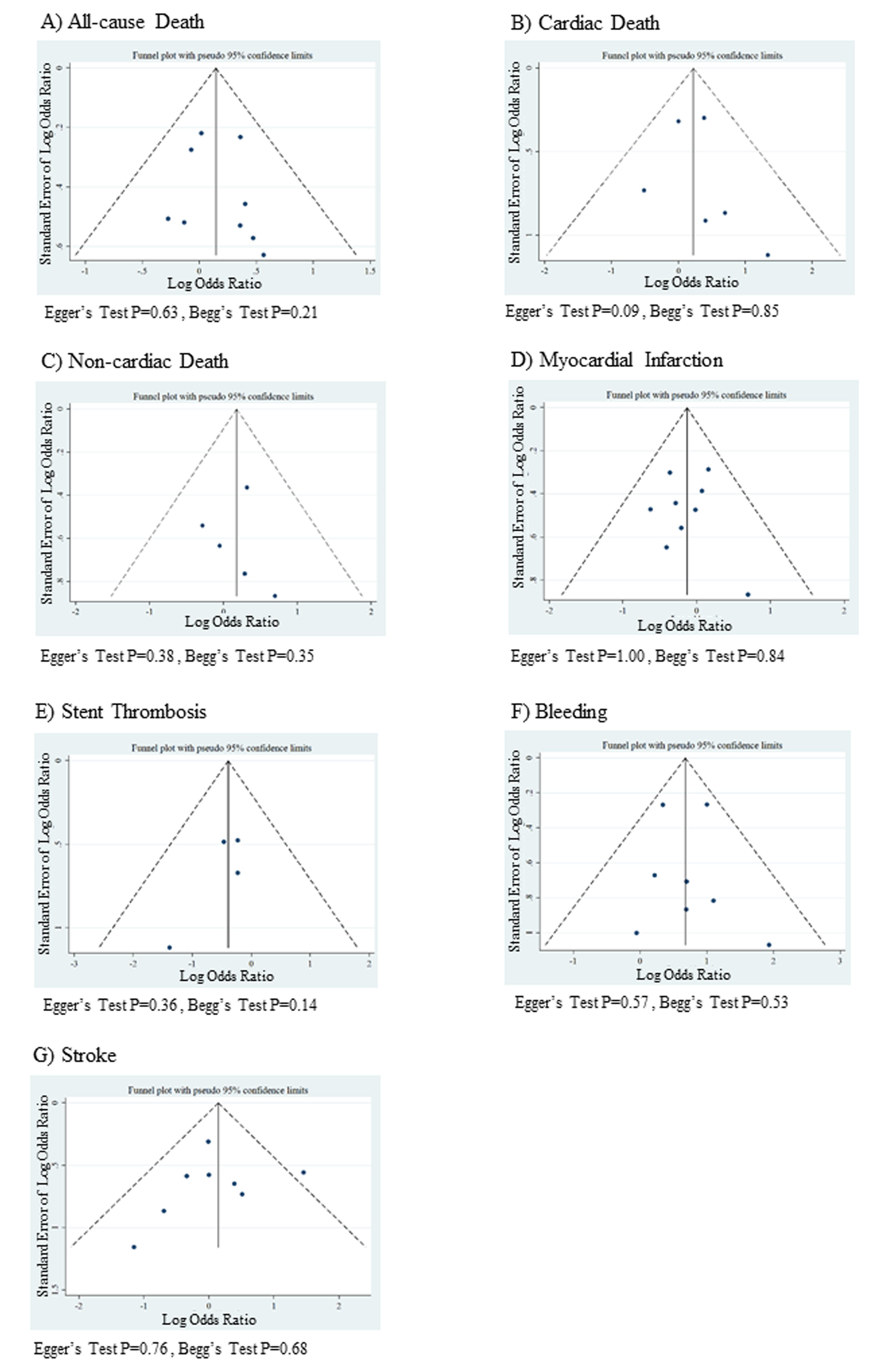

Supplement: S9 Fig — (TIF) [file pone.0174502.s009.tif]
