# Supplementary material for: Short versus prolonged dual antiplatelet therapy (DAPT) duration after coronary stent implantation: A comparison between the DAPT study and 9 other trials evaluating DAPT duration
Source: PLoS One. 2017 Sep 20;12(9):e0174502. doi: 10.1371/journal.pone.0174502 (PMC5607128; doi:10.1371/journal.pone.0174502)
Supplement: S1 Table — (DOCX) [file pone.0174502.s010.docx]

**Supplemental Table**

**S1 Table. List of the 10 trials analyzed in the current study.**

| Trials | Relative Reference / Major Inclusion and Exclusion Criteria |
| --- | --- |
| DAPT | Mauri L, Kereiakes DJ, Yeh RW, Driscoll-Shempp P, Cutlip DE, Steg PG, Normand SL, Braunwald E, Wiviott SD, Cohen DJ, Holmes DR, Jr., Krucoff MW, Hermiller J, Dauerman HL, Simon DI, Kandzari DE, Garratt KN, Lee DP, Pow TK, Lee PV, Rinaldi MJ, Massaro JM, the DAPT Study Investigators. Twelve or 30 Months of Dual Antiplatelet Therapy after Drug-Eluting Stents. The New England journal of medicine 2014;371:2155-66.  **Major Inclusion Criteria:**  · Undergoing percutaneous intervention with stent deployment  · Subject is “12-month clear” (free from myocardial infarction, stroke, repeat coronary revascularization, stent thrombosis, and moderate or severe bleeding and are compliant)  **Major Exclusion Criteria:**  · Index procedure stent placement with stent diameter <2.25 mm or >4.0 mm  · Planned surgery necessitating discontinuation of antiplatelet therapy within the 30 months after enrollment  · Current medical condition with a life expectancy of <3 years  · Subjects on warfarin or similar anticoagulant therapy  · Subject treated with both DES and BMS during the index procedure  · Subject switched thienopyridine type or dose within 6 months before randomization.  · Percutaneous coronary intervention or cardiac surgery between 6 weeks postindex procedure and randomization.  · Planned surgery necessitating discontinuation of antiplatelet therapy within the 21 months after randomization. |
| RESET | Kim BK, Hong MK, Shin DH, Nam CM, Kim JS, Ko YG, Choi D, Kang TS, Park BE, Kang WC, Lee SH, Yoon JH, Hong BK, Kwon HM, Jang Y, RESET Investigators. A new strategy for discontinuation of dual antiplatelet therapy: the RESET Trial (REal Safety and Efficacy of 3-month dual antiplatelet Therapy following Endeavor zotarolimus-eluting stent implantation). Journal of the American College of Cardiology 2012;60(15):1340-8.  **Major Inclusion Criteria:**  · Coronary artery disease including stable angina, unstable angina and acute myocardial infarction  · Patients with typical chest pain and objective evidences of myocardial ischemia on the electrocardiogram or positive functional studies consistent with reversible myocardial ischemia  · Reference vessel diameter of 2.5 to 4.0 mm by the visual estimation of operator assessment  **Major Exclusion Criteria:**  · Contraindication to anti-platelet agents & bleeding history within prior 3 months  · Prior history of the following presentations: 1) Cerebral vascular accidents (not including transient ischemic attack) 2) Peripheral artery occlusive diseases 3) Thrombo-embolic disease 4) Stent thrombosis  · Severe hepatic dysfunction (≥3 times normal reference values)  · Significant renal dysfunction (Serum creatinine >2.0 mg/dl)  · Significant leukopenia, neutropenia, thrombocytopenia, anemia, or known bleeding diathesis  · Cardiogenic shock  · Left ventricular ejection fraction <40%  · Life expectancy <3 year  · Acute ST-elevation myocardial infarction within 48 hours after onset of symptoms  · Left main disease requiring percutaneous coronary intervention  · Bifurcation lesion treated with a two-stent technique  · Target lesions with in-stent restenosis at the stented segment of DES or bare-metal stent  · Lesions with chronic total occlusion  · History of percutaneous coronary intervention with DESs  · Overlapped DESs (overlapping stenting was only allowed in the long-lesion subgroup) |
| OPTIMIZE | Feres F, Costa RA, Abizaid A, Leon MB, Marin-Neto JA, Botelho RV, King SB, 3rd, Negoita M, Liu M, de Paula JE, Mangione JA, Meireles GX, Castello HJ, Jr., Nicolela EL, Jr., Perin MA, Devito FS, Labrunie A, Salvadori D, Jr., Gusmao M, Staico R, Costa JR, Jr., de Castro JP, Abizaid AS, Bhatt DL, OPTIMIZE Trial Investigators. Three vs twelve months of dual antiplatelet therapy after zotarolimus-eluting stents: the OPTIMIZE randomized trial. Jama 2013;310(23):2510-22.  **Major Inclusion Criteria:**  · Clinical indication for PCI  · Lesion located in a native major epicardial vessel or a major side branch ≥2.50 mm (by visual estimation) or arterial conduit  · At least one stenosis ≥50% (by visual estimation)  · Coronary anatomy suitable for percutaneous treatment with E-ZES  **Major Exclusion Criteria:**  · STEMI presenting for primary or rescue PCI PCI with bare metal stent(s) in non-target lesion <6 mo prior to index procedure  · Previous treatment with any DES  · Scheduled elective surgery within 12 mo post index procedure  · Lesion located in saphenous vein graft  · DES in-stent restenosis |
| PRODIGY | Valgimigli M, Campo G, Monti M, Vranckx P, Percoco G, Tumscitz C, Castriota F, Colombo F, Tebaldi M, Fuca G, Kubbajeh M, Cangiano E, Minarelli M, Scalone A, Cavazza C, Frangione A, Borghesi M, Marchesini J, Parrinello G, Ferrari R, Prolonging Dual Antiplatelet Treatment After Grading Stent-Induced Intimal Hyperplasia Study Investigators. Short- versus long-term duration of dual-antiplatelet therapy after coronary stenting: a randomized multicenter trial. Circulation 2012;125(16):2015-26.  **Major Inclusion Criteria:**  · Patients undergoing elective, urgent, or emergent coronary angioplasty with intended stent implantation  · patients with chronic stable coronary artery disease or acute coronary syndromes, including non–ST-elevation and ST-elevation myocardial infarction  **Major Exclusion Criteria:**  · planned surgery within 24 months of percutaneous coronary intervention  · active bleeding or previous stroke in the past 6 months  · concomitant or foreseeable need for oral anticoagulation therapy  · life expectancy <24 months |
| EXCELLENT | Gwon HC, Hahn JY, Park KW, Song YB, Chae IH, Lim DS, Han KR, Choi JH, Choi SH, Kang HJ, Koo BK, Ahn T, Yoon JH, Jeong MH, Hong TJ, Chung WY, Choi YJ, Hur SH, Kwon HM, Jeon DW, Kim BO, Park SH, Lee NH, Jeon HK, Jang Y, Kim HS. Six-month versus 12-month dual antiplatelet therapy after implantation of drug-eluting stents: the Efficacy of Xience/Promus Versus Cypher to Reduce Late Loss After Stenting (EXCELLENT) randomized, multicenter study. Circulation 2012;125(3):505-13.  **Major Inclusion Criteria:**  · Patients were eligible for inclusion in the study if they had at least 1 lesion in a native coronary vessel with a reference diameter of 2.25 to 4.25 mm, stenosis of >50% by visual estimation, and evidence of myocardial ischemia such as stable angina, unstable angina, recent myocardial infarction, silent ischemia, a positive functional study, or reversible changes on ECG consistent with ischemia  **Major Exclusion Criteria:** · Exclusion criteria were myocardial infarction within 72 hours; severely compromised ventricular dysfunction (ejection fraction <25%) or cardiogenic shock; any stent implantation in the target vessel before enrollment; hemoglobin <10 g/dL or platelet count <100 000 per 1L; serum creatinine >=265.2mol/L (3.0 mg/dL) or dependence on dialysis; serious hepatic disease; major bleeding within 3 months or major surgery within 2 months; allergy to antiplatelet drugs, heparin, stainless steel, contrast agents, everolimus, or sirolimus; elective surgical procedure planned within <12 months; life expectancy <1 year; significant left main disease defined as stenosis of >50%; chronic total occlusion; true bifurcation lesions requiring a planned 2-stent strategy; or active participation in another clinical study. |
| SECURITY | Colombo A, Chieffo A, Frasheri A, Garbo R, Masotti M, Salvatella N, Oteo Dominguez JF, Steffanon L, Tarantini G, Presbitero P, Menozzi A, Pucci E, Mauri J, Cesana BM, Giustino G, Sardella G. Second Generation Drug-Eluting Stents Implantation Followed by Six Versus Twelve-Month - Dual Antiplatelet Therapy- The SECURITY Randomized Clinical Trial. Journal of the American College of Cardiology 2014;64:2086-97.  **Major Inclusion Criteria:**  · Inclusion criteria were symptoms of stable angina, as defined by Canadian Cardiovascular Society Classification, or unstable angina, as defined by Braunwald classification, or patients with documented silent ischemia, treated with at least 1 second-generation DES implanted in the target lesion in the past 24 h.  · Additional inclusion criteria were the presence of 1 or more de novo stenosis $70% in a native coronary artery, patient age over 18 years, no other DES implanted before the target procedure, and no bare-metal stent implanted in the 3 months before the target procedure.  **Major Exclusion Criteria:**  · Exclusion criteria were patients treated for saphenous vein graft, in-stent restenosis, unprotected left main coronary artery, ST-segment elevation myocardial infarction (MI) in the 48 h before the procedure, or non–ST-segment elevation MI in the previous 6 months; left ventricular ejection fraction #30%; known hypersensitivity to aspirin, thienopyridines, heparin, limus analogs, cobalt, chromium, nickel, molybdenum, or contrast media; history of significant thrombocytopenia with aspirin or thienopyridines; chronic kidney disease (creati-  nine >2 mg/dl); women during pregnancy or during lactation; active bleeding or significant risk of bleeding; uncontrolled hypertension; life expectancy <24 months; and any medical condition that could preclude follow-up, as defined in the protocol. There was no limit to the number of lesions that could be treated. |
| ITALIC/ITALIC+ | Gilard M, Barragan P, Noryani AA, Noor HA, Majwal T, Hovasse T, Castellant P, Schneeberger M, Maillard L, Bressolette EE, Wojcik J, Delarche N, Blanchard D, Jouve B, Ormezzano O, Paganelli F, Levy G, Sainsous J, Carrie D, Furber A, Berland J, Darremont O, Le Breton H, Lyuycx-Bore A, Gommeaux A, Cassat C, Kermarrec A, Cazaux P, Druelles P, Dauphin R, Armengaud J, Dupouy P, Champagnac D, Ohlmann P, Endresen KK, Benamer H, Kiss RG, Ungi I, Boschat JJ, Morice MC. Six-month versus 24-month dual antiplatelet therapy after implantation of drug eluting stents in patients non-resistant to aspirin: ITALIC, a randomized multicenter trial. Journal of the American College of Cardiology 2014;[Epub ahead of print] doi: 10.1016/j.jacc.2014.11.008.  **Major Inclusion Criteria:**  · eligible for percutaneous coronary intervention (PCI); implanted with at least 1 Xience V DES (Abbott Vascular Devices, Santa Clara, California); and all clinical situations excluding primary PCI for acute MI and treatment of the left main artery. Only treatment with Xience V was permitted.  **Major Exclusion Criteria:**  · Aspirin resistance was checked, and nonresponders were excluded from randomization.  · prior DES implantation within 1 year; known platelet level <100,000/ml or known hemorrhagic diathesis; oral anticoagulation therapy or abciximab treatment during hospital stay; contraindications to aspirin or clopidogrel (prasugrel or ticagrelor); major surgery within the preceding 6 weeks; evidence of active gastrointestinal or urogenital bleeding; severe liver failure; any surgery scheduled during the year after enrollment; or severe concomitant disease with <2 years’ life expectancy. |
| ISAR-SAFE | Schulz-Schupke S, Mehilli J, Laugwitz KL, Neumann FJ, Ten Berg JM, Adriaenssens T, Han Y, Merzljak B, Richardt G, Seyfarth M, Morath T, Maeng M, Zrenner B, Rifatov N, Jacobshagen C, Mudra H, Hodenberg E, Wohrle J, Kufner S, Hengstenberg C, Fischer M, Schmidt M, Dotzer F, Ibrahim T, Sick P, Nienaber CA, van't Hof AW, Kimura T, Witzenbichler B, Windecker S, Schunkert H, Kastrati A. Randomized, double-blind trial of 6 versus 12 months of dual antiplatelet therapy after DES implantation (ISAR-SAFE). AHA Scientific Session 2014;Available at (http://conference-cast.com/aha/media/AHA2014AM_12/LBCT.01/19352/19352.pdf), Accessed 1 December 2014.  Byrne RA, Schulz S, Mehilli J, Iijima R, Massberg S, Neumann FJ, ten Berg JM, Schomig A, Kastrati A, Intracoronary Stenting and Antithrombotic Regimen: Safety And Efficacy of Six Months Dual Antiplatelet Therapy After Drug-Eluting Stenting Investigators. Rationale and design of a randomized, double-blind, placebo-controlled trial of 6 versus 12 months clopidogrel therapy after implantation of a drug-eluting stent: The Intracoronary Stenting and Antithrombotic Regimen: Safety And EFficacy of 6 Months Dual Antiplatelet Therapy After Drug-Eluting Stenting (ISAR-SAFE) study. American heart journal 2009;157(4):620-4 e2.  **Major Inclusion Criteria:**  · Patients on clopidogrel at 6 months after DES  **Major Exclusion Criteria:**  · Clinical symptoms or signs of ischemia and/or angiographic lesions requiring revascularization  · acute bleeding: bleeding disthesis; history of intracranial bleeding  · STEMI and NSTEMI during 6 month after DES  · Previous stent thrombosis  · DES in left main coronary artery at index intervention  · Oral coagulation  · Planned major surgery within the next 6 month with need to discontinue antiplatelet therapy |
| DES LATE | Lee CW, Ahn JM, Park DW, Kang SJ, Lee SW, Kim YH, Park SW, Han S, Lee SG, Seong IW, Rha SW, Jeong MH, Lim DS, Yoon JH, Hur SH, Choi YS, Yang JY, Lee NH, Kim HS, Lee BK, Kim KS, Lee SU, Chae JK, Cheong SS, Suh IW, Park HS, Nah DY, Jeon DS, Seung KB, Lee K, Jang JS, Park SJ. Optimal duration of dual antiplatelet therapy after drug-eluting stent implantation: a randomized, controlled trial. Circulation 2014;129(3):304-12.  **Major Inclusion Criteria:**  · Patients were eligible for participation in the study if they had undergone implantation with drug-eluting stents at least 12 months before  enrollment, had not had a major adverse cardiovascular event (myocardial infarction, stroke, or repeat revascularization) or major bleed-  ing since implantation, and were receiving dual antiplatelet therapy at the time of enrollment.  **Major Exclusion Criteria:**  · contraindications to the use of antiplatelet drugs, concomitant vascular disease that required the long-term use of clopidogrel or  other established indications for clopidogrel therapy (eg, recent acute coronary syndrome), and noncardiac coexisting conditions with a life  expectancy of <1 year |
| ARCTIC-Interruption | Collet JP, Silvain J, Barthelemy O, Range G, Cayla G, Van Belle E, Cuisset T, Elhadad S, Schiele F, Lhoest N, Ohlmann P, Carrie D, Rousseau H, Aubry P, Monsegu J, Sabouret P, O'Connor SA, Abtan J, Kerneis M, Saint-Etienne C, Beygui F, Vicaut E, Montalescot G, for the ARCTIC investigators. Dual-antiplatelet treatment beyond 1 year after drug-eluting stent implantation (ARCTIC-Interruption): a randomised trial. Lancet 2014;384:1577-85.  **Major Inclusion Criteria:**  · planned DES implantation  **Major Exclusion Criteria:**  · ST-elevation myocardial infarction,  · planned use of glycoprotein IIb/IIa inhibitors, chronic anticoagulation treatment, or bleeding diathesis  · occurrence of any ischaemic event of the primary endpoint or any event of the primary safety endpoint during the first year of follow-up after the randomization in the first phase of the study, any new revascularisation needing DAPT extension  · any contraindication to aspirin continuation such as bleeding gastrointestinal ulcer, or aspirin resistance. |
