# Supplementary material for: Short versus prolonged dual antiplatelet therapy (DAPT) duration after coronary stent implantation: A comparison between the DAPT study and 9 other trials evaluating DAPT duration
Source: PLoS One. 2017 Sep 20;12(9):e0174502. doi: 10.1371/journal.pone.0174502 (PMC5607128; doi:10.1371/journal.pone.0174502)
Supplement: S2 Table — (DOCX) [file pone.0174502.s011.docx]

**Supplemental Table**

**S2 Table. Cochrane’s collaboration tool for assessing risk of bias among included studies.**

| **Trials** | **DAPT** | **RESET** | **OPTIMIZE** | **PRODIGY** | **EXCELLENT** | **SECURITY** | **ITALIC**  **/ITALIC+** | **ISAR-SAFE** | **DES LATE** | **ARCTIC**  **-Interruption** |
| --- | --- | --- | --- | --- | --- | --- | --- | --- | --- | --- |
| Sequence generation | Yes | Yes | Yes | Yes | Yes | Yes | Yes | Yes | Yes | Yes |
| Allocation concealment | Yes | Yes | Yes | Yes | Yes | Yes | Yes | Yes | Yes | Yes |
| Blinding of participants and personnel | Yes | No | No | No | No | No | No | Yes | No | No |
| Incomplete outcome data | Low | Low | Low | Low | Low | Low | Low | Low | Low | Low |
| Selective outcome reporting | Low | Low | Low | Low | Low | Low | Low | Low | Low | Low |
| Other sources of bias | Low | Low | Low | Low | Low | Low | Low | Low | Low | Low |
| Risk of bias | Low | Low | Low | Low | Low | Low | Low | Low | Low | Low |

For sequence generation, allocation concealment, and blinding of participants and personnel, risk of bias were assessed as “Yes” or “No”. Regarding incomplete outcome data, selective outcome reporting, other sources of bias, and risk of bias were evaluated as “Low” or “High”
